# Molecular determinants of AR–enhancer interaction and cistrome reprogramming in prostate cancer progression

**DOI:** 10.1530/ERC-25-0529

**Published:** 2026-06-15

**Authors:** Rayzel C Fernandes, Kyle K Greenland, Moray J Campbell, Charlotte L Bevan

**Affiliations:** ^1^ Division of Cancer, Imperial Centre for Translational & Experimental Medicine, Imperial College London, London, UK; ^2^ Barbara Ann Karmanos Cancer Institute, Department of Oncology, Wayne State University, Detroit, Michigan, USA

**Keywords:** prostate cancer, androgen receptor, reprogramming, cistrome

## Abstract

The androgen receptor (AR) is a key transcription factor in prostate cancer (PCa), whose enhanced and altered functions are known drivers of cancer progression. A key aspect of this is reprogramming of the AR cistrome, which consists of genome-wide enhancer-binding sites through which AR regulates gene expression. The magnitude and biological impact of the AR cistrome are impacted by the AR itself, including the responses to ligand, as well as the organization of the associated DNA response elements, and availability of pioneer factors, cofactors, and noncoding RNAs, all of which contribute to a functional transcription complex. In this review, we will examine, in the context of PCa progression, the factors that affect the binding of AR and its interacting partners at enhancers, with a focus on AR cistrome reprogramming. We also discuss the clinical utility of targeting the AR–enhancer nucleoprotein complex and the potential of using the AR cistrome as a prognostic tool.

## The paradigm of integrated AR gene transactivation

The majority of the human genome consists of nonprotein-coding regions, which are central to the regulation of the smaller proportion of the genome that contains protein-coding genes. More specifically, it is estimated that these noncoding regulatory regions compose approximately 8% of the mappable human genome (1), which is appreciably larger than the proportion of the genome that is protein-coding (∼1%). Regulatory elements have classically been defined as regions that modulate gene expression by initiating (promoters) and increasing or decreasing the rate of (enhancers and silencers, respectively) transcription (2, 3). Enhancers were discovered in the 1980s, when an SV40 viral DNA sequence was found to enhance the expression of a linked gene over long distances and irrespective of orientation (2). Consequently, enhancer elements were discovered within mammalian genomes and found to act in a tissue-specific manner, as in the case of the mouse IgG heavy chain gene (4). While early enhancer discoveries were based on functional reporter-based assays, subsequent high-throughput genomic sequencing and biochemical assays (such as work from the ENCODE project) led to the identification of more than a million enhancer-like elements in mammalian cells and tissues. Enhancer elements are typically a few hundred base pairs in length and consist of clusters of transcription factor-binding sites that are recognized by transcription factors (TFs) to drive spatial-, temporal-, and cell-specific gene expression programs (5). More recently, clusters of enhancers, termed super-enhancers, have been identified, which seem to mediate the functions of multiple TFs to govern major cell lineage decisions (6).

Changes that affect enhancer structure and function contribute to a range of diseases. In particular, enhancer dysfunction is a key player in hormone-dependent malignancies, such as prostate cancer, which are driven by hormone-activated TFs that regulate gene expression primarily through binding to recognition sequences within enhancers. In prostate cancer, the androgen receptor (AR) is a hormone-activated TF that significantly impacts tissue homeostasis. At the cellular level, the AR is activated in response to binding of its ligands, the hormones testosterone or dihydrotestosterone (DHT), which induces conformational changes that support receptor dimerization, translocation to the nucleus, DNA binding, and protein–protein interactions. In the nucleus, AR dimers engage with androgen response elements (AREs) on target DNA, which are mainly within distal cis-regulatory enhancers and, to a lesser extent, in proximal promoters and intronic regions (7, 8). This set of genome-wide AR-bound sites constitutes the ‘AR cistrome’ and is a critical factor in prostate organogenesis, and prostate cancer (PCa), disease progression, and prognosis. Response elements for hormone-dependent nuclear receptors comprise two hexamer half-sites separated by a spacer (9). The canonical ARE motif consists of an inverted 5′-AGAACA-3′ repeat with a 3 bp spacer (10), although the majority of experimentally identified androgen receptor-binding sites (ARBS) are imperfect AREs with several base pairs deviating from the canonical sequence or consisting of ARE half-sites (11, 12). More specifically, in PCa cell lines, the consensus sequence accounts for approximately 0.1% of ARBS, whereas deviations of 1–3 bps from the consensus make up approximately 35% of sites and half-sites constitute the majority at ∼65% (11). Degeneracy from the consensus ARE impacts AR-binding affinity and tissue-specific transcriptional responses (11). Indeed, weaker affinity degenerate sites have been shown to require cooperation with other TFs and regulatory proteins and can be activated to drive oncogenic transcription in PCa (11, 13).

At any point in time, only a small fraction of the genome exists as euchromatin, i.e. nucleosome-depleted regions accessible to TFs and/or other proteins, whereas the majority exists as nucleosome-dense, compact heterochromatin (14, 15). Cell state transitions that occur during development – and also in cancer progression – require relaxing of heterochromatin to allow activation of lineage-specific and other gene networks (15). The prevailing theory suggests that this is undertaken by pioneer factors, a subset of TFs that have the ability to bind compacted chromatin, inducing an open conformation so that non-pioneering TFs, plus cofactors, can then access and bind these regions (16). However, this process is likely to be more nuanced and context-dependent (17). In PCa, ARBS that are gained during progression are already accessible and bound by the pioneer factors forkhead box protein A1 (FOXA1) and homeobox B13 (HOXB13) in normal and primary tumor tissue (18), suggesting that factors besides accessibility drive disease-associated AR cistromic changes.

Cofactors are proteins that are classically defined as those that, while not binding DNA directly, interact with TFs and either increase (coactivators) or decrease (corepressors) the rate of transcription without affecting the basal transcription rate (19). A large fraction of cofactors are epigenetic regulators that act through mechanisms such as nucleosome remodeling and the regulation of histone modifications. Nucleosome remodelers such as human switch/sucrose non-fermentable (SWI/SNF), chromodomain helicase DNA-binding (CHD), and imitation SWI (ISWI) are multi-subunit complexes that use energy derived from ATP hydrolysis to remove or reposition nucleosomes, thus providing access to regulatory sites (20). This provides a biologically plausible link to the also large group of histone modifiers that deposit or remove chemical groups on histone tails, which alter their charge and, thus, influence the way these proteins interact with DNA or chromatin-binding proteins and thereby influencing accessibility (21). These marks are most commonly acetyl and methyl groups – while acetylation of histones is generally associated with permissive chromatin, the effect of methylation depends on the histone residue modified (21, 22). Marks deposited, or ‘written’, by histone modifiers are, in turn, recognized or ‘read’ by proteins containing reader domains such as the plant homeodomain (PHD) and bromodomain (BRD), which recognize methylated lysine/arginine residues and acetylated lysine residues on histones, respectively (23). Reader domains link and recruit other proteins within the enhancer complex to drive downstream transcriptional events (24). Other proteins, such as general TFs, architectural proteins (cohesins and CTCF), and RNA polymerase II, are also essential components of AR–enhancer complexes. These components all contribute to the established model of transactivation, whereby AR binds to target enhancers and regulatory regions that are made accessible by the interplay between ATP-dependent chromatin remodelers and pioneer factors that stabilize nucleosome positioning, and, in turn, recruit cofactors to further enhance and stabilize chromatin accessibility and drive transcriptional activity (25).

In parallel with proteomic factors, noncoding enhancer RNAs and long noncoding RNAs also associate with AR–enhancer complexes to further regulate function. ncRNAs are known to interact with DNA-binding proteins and chromatin, and emerging evidence suggests these molecules are essential for recruiting regulators to enhancers and for the formation of enhancer–promoter interaction (26, 27). Long-range enhancer–promoter interactions are critical in gene regulation, given that enhancer elements are generally located distal to their target genes (7). These interactions are achieved through folding of the genome into loops and mediated by nucleo–protein complexes at enhancers and promoters (28, 29). Perturbations affecting components of AR–enhancer complexes in PCa can, therefore, influence three-dimensional (3D) genome organization (30, 31).

Therapeutically, the AR axis is very significant. Low- and intermediate-risk localized PCa is managed with active surveillance or treated with interventions such as surgery or radiation (32). High-risk localized PCa, locally advanced PCa, metastatic disease, and recurrent disease are treated with combinations of surgery, radiation, and androgen deprivation therapy (ADT) (32). ADT aims to reduce levels of circulating androgens and, thus, minimize AR-mediated PCa proliferation (33). While this is initially successful, most patients eventually relapse to an ADT recurrent state termed castration-resistant PCa (CRPC), in which the cancer continues to grow and spread despite low levels of androgens (34). The AR signaling axis persists in CRPC and is targeted with androgen receptor pathway inhibitors (ARPIs), such as enzalutamide, apalutamide, darolutamide, and abiraterone (35, 36, 37). More recently, ARPIs have been shown to improve overall survival when used in combination with ADT and chemotherapeutic agents, such as docetaxel, for metastatic castration-sensitive PCa (38, 39, 40). Resistance to ARPIs also eventually emerges and is associated with poor prognosis. The emergence of resistance to AR-directed therapy in PCa has been attributed to alterations in the AR signaling pathway, with CRPC tumors from patients exposed to ADT and ARPIs demonstrating a higher frequency of AR-related alterations compared to treatment naïve patients. For instance, AR amplifications have been observed in up to 70% of CRPC (41, 42), AR mutations occur in 10–30% of CRPC treated with antiandrogens (43), and AR splice variants have been detected in 75–88% of CRPC tumors (44, 45). In contrast, treatment naïve primary tumors have low to no AR-related alterations (44, 45).

**Figure 1:**
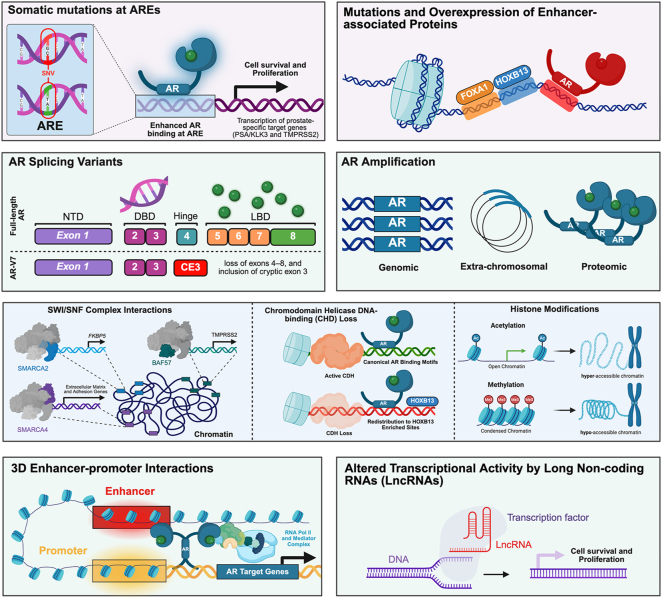
Factors influencing altered AR binding and cistrome reprogramming in prostate cancer. The AR cistrome can be altered by a multitude of factors, from somatic mutations in AREs to overexpression of enhancer-associated proteins, as well as AR amplification and splice variants, changes in epigenetic coregulators, disruption of 3D genomic organization, and alterations in transcriptional activity by long noncoding RNAs (lncRNAs).

Enhancer sequences as well as components of the AR–enhancer complex, such as pioneer factors, cofactors, and noncoding RNAs, are often critical regulators of AR-mediated transcriptional programs, determining which target genes are expressed in a given context. Importantly, studies have shown that their dysregulation, either via mutations or changes in expression levels or activity, impacts AR transcriptional activity and consequently contributes to all stages of prostate cancer, including metastases and the development of resistance to AR targeting therapies (46, 47, 48, 49). In this review, we examine, in the context of PCa progression, the factors that affect binding of AR and its interacting partners at enhancers (Fig. 1), with a focus on AR cistrome reprogramming. We also discuss the clinical utility of targeting the AR–enhancer nucleoprotein complex and the potential of using the AR cistrome as a prognostic tool.

## AR cistrome and its reprogramming in PCa

While potential AREs likely occur millions of times in the human genome, only a fraction are bound by AR and constitute the AR cistrome in a given context (7, 11, 50, 51). This differential access and binding to AREs are important for normal biological processes. For example, during development, the AR cistrome has been observed to be distinct in male and female rodent tissues, indicating a role in sexual dimorphism (52). The AR cistrome has also been shown to be essential for prostate luminal differentiation and to distinguish between luminal and basal prostatic cells (53). Perhaps reflecting this flexibility over the choice of binding site, it is now well established that the AR cistrome, along with its associated transcriptome, undergoes dynamic changes during PCa progression.

Profiling the AR cistrome in normal and primary prostate tumor tissue has demonstrated that there are shared as well as unique ARBS between the two conditions (51, 54, 55). Genes associated with ARBS unique to tumors can predict tumor aggressiveness and patient survival (51, 54). A subsequent study also demonstrated that the AR cistrome is reprogrammed across clinical states from normal prostate epithelium to primary PCa and subsequently metastatic disease, with AR moving to sites associated with developmental programs, during progression (18). Changes to the AR cistrome also contribute to androgen independence. For example, in androgen-independent cell line models, where the AR is active in the absence of androgen stimulation, the AR cistrome is associated with higher expression of cell cycle and M-phase genes compared to androgen-dependent cell lines (31). Other studies using patient samples have shown that there are differences when comparing ARBS between castration-resistant PCa and untreated PCa (56) and identified distinct ARBS profiles between primary PCa and tumors with acquired therapy resistance (57). Consistent with these reports, changes in AR-binding profiles have also been observed in the transition from localized hormone-sensitive PCa to metastatic CRPC (18). Furthermore, in transition toward a therapy-resistant state, the AR cistrome has been shown to be redirected toward pathways that promote stem cell plasticity and neuronal processes (58). Overall, these reports suggest that the AR cistrome plays a key role in PCa progression, from disease initiation to the acquisition of therapy resistance. These changes to the AR cistrome are driven by several upstream factors that influence binding site selection.

## Factors contributing to altered AR–enhancer binding and cistrome reprogramming in PCa

### Genomic alterations at AR-regulated enhancers

Genetic alterations, particularly somatic mutations such as copy number variations (CNVs) and single-nucleotide variations (SNVs), impact enhancer function, TF binding, and gene transcription. In case of PCa, these alterations can be drivers of disease progression, with, for example, amplification of an enhancer for the AR gene (chrX: 66117800-66128800, hg19) being a commonly occurring CNV in metastatic castration-resistant disease but is very rare in primary PCa (59, 60). This alteration has been reported to occur in approximately 70–80% of metastatic CRPC when compared to less than 2% of primary prostate cancer (41, 59). This enhancer is bound by the AR along with other TFs and is essential for viability and castration resistance as it upregulates expression of the AR protein (60, 61).

ARBS have also been shown to have higher rates of somatic mutations in PCa compared to other cancers or normal tissue (62, 63). This is suggested to be a consequence of AR occupancy, with AR potentially inducing DNA damage when bound to chromatin or preventing access by the repair machinery (62). A number of SNVs have also been observed to be significantly enriched within ARBS in metastatic PCa (64). SNVs within ARBS can impact PCa progression by altering AREs, which, in turn, affects AR-binding affinity and enhancer activation (65, 66, 67, 68). SNVs can, thus, either augment or impair AR-regulated enhancer activity.

### AR amplification and splice variants

Progression of PCa to a therapy resistance state is often associated with gain or amplification of the *AR* gene, AR protein overexpression, and the expression of constitutively active AR variants (AR-Vs) (41, 42, 69, 70). AR gene amplification and the expression of splice variants are low in hormone-naive PCa but significantly increase following ADT or treatment with ARPIs (41, 42, 45, 71). AR overexpression sensitizes cancer cells to low levels of androgens and has been shown to promote androgen-independent growth (70, 72). This is likely due to an impact on the cistrome, with higher AR expression associated with a higher number of ARBS and stronger binding, on stimulation with low levels of androgen (73). Modulation of AR levels by itself, in the absence of factors like FOXA1 and HOXB13, was unable to alter AR binding (51), highlighting the role of multiple players in driving cistromic changes during PCa progression.

High AR copy number and expression in CRPC has recently also been shown to be attributable in some cases to the presence of the AR locus on extrachromosomal circular DNA (ecDNA) (74, 75). AR-amplifying ecDNA has been reported to be more common in ARPI-resistant CRCP compared to ARPI naïve CRPC (76). Consistent with this, metastatic CRPC patients with AR-amplifying ecDNA have been suggested to be resistant to ARPIs (74). Interestingly, AR from ecDNA appears to be transcribed into novel mRNA variants, which could give rise to AR-Vs and contribute to therapy resistance (75).

AR-Vs, which can regulate transcription in a ligand-independent manner, are generated by alternative splicing of the AR mRNA (e.g. AR-V1, 4, 5, 6, 7, 9) or by genetic rearrangements of the AR gene (e.g. ARv567es) and usually have an N-terminal domain (NTD) and DNA-binding domain (DBD) similar to the full-length AR (AR-FL) but have a short variant-specific peptide instead of the C-terminal ligand-binding domain, which can impact interactions with coregulators (77, 78). While more than 20 AR-Vs have been reported in PCa cell lines and tissues so far (77), AR-V7 and ARv567es are the most widely studied in the context of the AR cistrome. Despite having a DBD similar to the AR-FL and sharing common binding sites, some studies have shown both AR-V7 and ARv567es to have unique subsets of binding sites (79, 80, 81, 82), although it is important to note that other studies suggest differences in binding sites to be minimal (83). In the case of AR-V7, the distinct cistrome was shown to regulate oncogenes in pathways contributing to CRPC, therapeutic resistance, metastases, and poor survival (80, 81, 84, 85). In addition to upregulating oncogenes, AR-V7 also contributes to CRPC by repressing the expression of tumor-suppressor genes (83). The reported divergence between AR-FL and AR-V7 cistromes appears to be, in part, due to a preference for binding location, with AR-V7 favoring binding to proximal promoter sites compared to AR-FL (86). Pioneer factors such as FOXA1 and coregulators also likely play a role in the divergence between AR-FL and AR-V cistromes and transcription programs (87, 88, 89).

### Proteome at AR-regulated enhancers in PCa

#### Pioneer factors

AR recruitment to enhancer regions is dictated by several pioneer factors, including forkhead box protein A1 (FOXA1), homeobox B13 (HOXB13), and GATA-binding factor 2 (GATA2). FOXA1 is a lineage-defining factor that is essential for prostate morphogenesis but also drives cell-type-specific transcriptional programs in breast and prostate cancers (90, 91). Specifically, FOXA1 is differentially recruited to enhancers in breast and prostate cancer cells, where it subsequently recruits ER and AR, respectively (90). At the genome-wide level, approximately 70% of ARBS were found to overlap with FOXA1-binding sites, and conversely, approximately 24% of FOXA1 sites were shared with ARBS (92).

Changes in cellular levels of FOXA1 profoundly alter the AR cistrome, with depletion of FOXA1 redistributing AR, as well as significantly increasing the total number of ARBS (92, 93, 94). Similarly, loss of FOXA1 induces androgen-independent binding of the AR to chromatin and has been shown to promote progression to hormone independence in cell line models (94). Since FOXA1 is a pioneer factor for AR, this apparently contradictory increase in ARBS was proposed to be a result of sites that were masked by FOXA1 and, thus, available to the AR on its depletion (92). A later study proposed that when FOXA1 is low, AR freely binds to AREs in the genome, whereas when present at high levels, FOXA1 causes indiscriminate opening of chromatin leading to nonspecific AR binding (94). When both proteins are in equilibrium, FOXA1 directs AR to AREs, which also overlap with the correlated motif (94). FOXA1 is also frequently mutated in PCa, at ∼4% in primary PCa and 12% in metastatic CRPC, with these mutations affecting its AR pioneering role (47). FOXA1 mutants vary in their ability to interact with AR and AR-regulated enhancers depending on the type of mutation present (47, 95, 96, 97). The majority of FOXA1 mutations are missense or indels (class 1 mutations) located within the forkhead DNA-binding domain (FKHD) that affect binding to DNA and are enriched in primary prostate tumors (47, 97). Recently, FOXA1 class 1 mutations have been found to pioneer AR neo-enhancers that are enriched for FOXA1 and chimeric FOXA1:AR half-motifs compared to the normal AR cistrome, which is enriched for palindromic AREs (98).

Similar to FOXA1, HOXB13 has been shown to be important for cellular response to androgens (99). HOXB13 regulates the interaction of both the AR and its coregulators to chromatin, but its effect on AR target gene expression can be negative or positive and has been proposed to depend on the type of interaction between HOXB13 and AR (99). When coregulated gene enhancers have both an ARE and HOXB13 recognition element present, they are likely to be activated by androgens. Consistent with this, HOXA1 has been shown to increase accessibility at binding sites that also contain AR motifs (100). In the case of the subset of HOXB13-repressed AR targets, this has been proposed to occur due to HOXB13 binding to the AR DBD and inhibiting its interaction with DNA (99, 100). HOXB13 also has AR-independent functions that can promote androgen-independent growth in AR-positive and AR-negative PCa (100, 101). GATA2 has similarly been shown to have the ability to pioneer AR binding to enhancers (102). This factor has been reported to bind AR target enhancers prior to hormone stimulation, to establish accessible chromatin, and contributes to the formation of enhancer-promoter loops (102).

**Figure 2:**
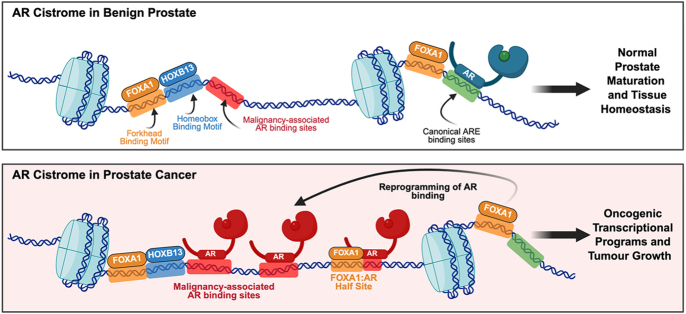
AR cistrome in normal and prostate cancer cells. Reprogramming from normal to malignant state is driven by activation of malignancy-associated binding sites by pioneer factors, such as FOXA1, HOXB13, and binding to low-affinity half-androgen response elements (half-AREs).

Each pioneer factor regulates multiple TFs, and selective action on each TF is likely influenced by other pioneer factors and coregulators. In case of the AR, FOXA1 has been shown to work in concert with the HOXB13 pioneer factor and other regulators to dysregulate the AR cistrome during PCa progression (Fig. 2). Both FOXA1 and HOXB13 have been observed to extensively colocalize with the AR at tumor-enriched ARBS in clinical PCa samples (51). This colocalization is functionally important – when both factors are concomitantly overexpressed in a nonmalignant prostate epithelial cell line, AR is reprogrammed to tumor-specific ARBS and away from ARBS that are specific to normal prostate tissue (51). In the same cell line, overexpression of FOXA1 and HOXB13 individually redistributes AR but not to the same extent as overexpression of both genes together (51). Subsequent work has shown that FOXA1 and HOXB13 are already present at sites that AR redistributes to during disease progression (18). As observed with HOXB13, GATA2 colocalizes with FOXA1 and AR on chromatin and is required for recruitment of coactivators to AR-regulated enhancers (102, 103). More recently, FOXA1 has been proposed to recruit HOXB13 and GATA2 to regulate the AR cistrome (104). In case of HOXB13, two germline variants, G84E and X285K, have been reported as associated with PCa susceptibility and aggressive disease (105, 106); however, their role, if any, in regulating AR activity is unknown. To date, GATA2 mutations have not been reported in the context of PCa and AR activity.

Besides regulating AR-FL, pioneer factors can contribute to changes in the AR cistrome in PCa by regulating the activity of AR-Vs. FOXA1, for example, has been found to coregulate AR-V7 target genes that have a pro-proliferative role in CRPC (87). HOXB13 has likewise been shown to coregulate oncogenic genes with AR-V7 that contribute to CRPC progression, in both cell lines and patient tissues (80). GATA2 has similarly been shown to regulate expression of a subset of AR-V target genes (107).

#### Epigenetic coregulators

Two families of nucleosome remodelers – SWI/SNF and CHD have been reported to play a role in regulating the AR cistrome. The SWI/SNF subunit SMARCA4, for example, has been reported to be recruited by activated AR, colocalizing with the majority of ARBS and likely affecting the function of FOXA1 and HOXB13 (108). Inhibition of SMARCA4, as well as other SWI/SNF subunits SMARCA2 and PBRM1, leads to chromatin compaction, loss of enhancer-promoter looping, and downregulation of AR- and FOXA1-regulated genes (109). Interestingly, disruption of the SWI/SNF complex results in partial loss of expression of AR and FOXA1 themselves, as a result of the collapse of enhancer-promoter looping driving their expression (109). Increased expression of SWI/SNF components is generally oncogenic in PCa, whereas the CHD family tends to be a tumor suppressor. CHD1, for example, is frequently deleted in PCa, with its loss resulting in prostate tumor formation (46). This is likely due to reprogramming of AR to oncogenic HOXB13-enriched sites that do not require CHD1 remodeling activity, following loss of CHD1 (46).

Histone-modifying complexes have been intensively investigated in PCa and have revealed that multiple components are altered. Enhancer of zeste homolog 2 (EZH2) is a histone methyltransferase that is frequently overexpressed in PCa (110). As the catalytic subunit of the polycomb repressor complex 2 (PRC2), EZH2 is normally responsible for repressing tumor-suppressive genes by trimethylation of histone H3 lysine 27 at their promoters (111). EZH2, however, has noncanonical roles independent of the PRC2 complex, which includes acting as a coactivator for AR and AR-V7 (88, 89). In this context, EZH2 cooperates with AR and AR-V7 to drive transcription of a subset of oncogenes that correlate with poor outcomes in PCa (89). Consistent with this, under conditions of high AR expression but castrate levels of androgens, EZH2 is involved in AR reprogramming to activate a transcriptional program that correlates with poor clinical outcome in patients (112). EZH2-mediated AR reprogramming is also a factor in resistance to AR-directed therapy. Specifically, AR can bind with EZH2 at sites associated with active gene transcription in enzalutamide-resistant cell lines and patient tumors (58). Here, EZH2 appears to interact with AR as part of a noncanonical PRC2 complex (58).

The histone mono- and di-methyltransferase nuclear receptor-binding SET domain protein 2 (NSD2) has also been shown to contribute to oncogenic AR reprogramming (48). NSD2 is a non-DNA-binding coregulator that is not expressed in normal prostate epithelial cells but increases with cancer progression (113). This coregulator enables binding of AR at chimeric FOXA1:AR half-motifs, with motifs for HOXB13 and ETS also found in close proximity to NDS2-dependant AR half-sites (48). Such chimeric half-motifs have been found to constitute approximately two-thirds of PCa AR cistrome, and NSD2 has, thus, been suggested to assist with reprogramming of the AR to these motifs by FOXA1, HOXB13, and ETS (48). Depletion of NSD2 results in loss of AR from approximately 65% of the tumor cistrome (48). Its paralog NSD1, which is upregulated in metastatic compared to primary PCa (114), has also been shown to enable oncogenic AR activity in PCa (48).

As another example of dysregulated histone modifiers affecting the AR cistrome, the PCa overexpressed protein arginine methyltransferase 1 (PRMT1), which catalyzes asymmetric dimethylation of H4R3 to open chromatin and is required for binding AR to its canonical target sites (115). Specifically, PRMT1 is associated with AR occupancy at lineage-specific enhancers and the tumor-specific AR cistrome (115). Although the mechanism behind this is unclear, it has been proposed to be related to PRMT1’s methyltransferase activity and its ability to interact with and modify other members of the AR transcriptional complex (115). PRMT1 also regulates the expression and stability of AR and AR-V7 (115).

Histone acetylases, such as members of the p160/SRC family, lysine acetyltransferase (KAT) family, and p300/CBP, have been shown to be oncogenic in PCa (116, 117, 118). Although members of the p160 family have weak acetylation activity, they recruit other acetylases, such as p300/CBP and PCAF, to AR-regulated regions (119). p300, for example, has also been shown to be important for regulating AR activity at multiple target sites in PCa, as evidenced by its colocalization with ∼48–83% of AR-bound sites (120, 121). AR–p300 co-occupancy is characterized by elevated histone H2B N-terminal acetylation, higher chromatin accessibility, increased cofactor occupancy, the formation of super-enhancers, and higher transcriptional output (121).

As an example of an epigenetic reader affecting the AR cistrome, tripartite motif-containing 24 (TRIM24) is a reader containing both PHD and bromodomain, along with a motif that enables binding to the AR (122). This allows it to bind acetylated histones and AR simultaneously, thereby anchoring AR to chromatin even under low-hormone conditions. TRIM24 expression increases during progression from primary PCa to recurrent and CRPC disease, where it enables the expression of AR-responsive and cell cycle-associated genes under hormone-starved conditions compared to hormone-stimulated conditions (122).

#### Other proteomic regulators of the AR cistrome

A number of other proteins affect the AR cistrome via mechanisms that are not epigenetic and/or related to pioneering activity. The most well-known of these belongs to the E-26 transformation-specific (ETS) family of TFs, which includes 28 members in humans, some of which are translocated and form gene fusion proteins in PCa (123). ETS gene fusions are driver mutations, occurring in the majority of PCa (124). The most common of these is a fusion between the promoter of the AR target gene TMPRSS2 and the coding region of the ETS family member ERG (125). This rearrangement occurs in approximately 50% of prostate tumors and leads to androgen-driven overexpression of the ERG protein (124). This translocation is probably highly impactful as ERG itself is an AR coregulator, able to interact with and regulate AR binding to DNA (13). Increased expression of ERG has been shown to alter the AR cistrome to increase the number of ARBS (126). In particular, ERG-mediated changes to the AR cistrome appear to partially restore AR signaling in tumors with loss of the phosphatase and tensin homolog (PTEN) tumor suppressor (126). Another ETS family member, ETV1, has also been shown to positively regulate the AR cistrome in PTEN-deficient tumors (126). ERG/AR shared sites can be co-bound by p300, which results in enhanced transcriptional activation (121).

As other examples of AR cistrome regulators, the single-minded homolog 2 (SIM2) TF, which shows increasing expression with PCa progression, affects chromatin occupancy at a fraction of ARBS (108). SIM2-associated ARBs are independent from those affected by FOXA1 (108). The mediator complex subunit 1 (MED1) interacts with AR and is recruited to ARBS upon androgen stimulation, where it is proposed to stabilize AR complexes and promote looping interactions with target promoters (127). Zinc finger X-linked (ZFX) partners with AR-V7 to activate a unique subset of genes separate from those regulated by AR-V7/FOXA1 and full-length AR (82).

### Noncoding RNAs as regulators of the AR cistrome

In the past decade, enhancers were found to be transcribed by RNA polymerase II to produce noncoding RNA (ncRNA) molecules termed enhancer RNAs (128, 129). The majority of eRNAs are highly unstable and proposed to be less than 500 bp in length, bidirectionally transcribed and non-polyadenylated, although longer (up to 4 kb), unidirectional, polyadenylated eRNAs have also been identified (129, 130). CAGE (cap analysis of gene expression) and other nascent RNA sequencing techniques have, in recent years, detected thousands of eRNAs. In prostate cancer, eRNAs have been shown to be induced by the activation of the AR in CRPC cells, are expressed from ARBS, and participate in AR-driven gene expression (26, 131, 132). Specifically, eRNAs appear to be transcribed from a fraction of ARBS that are proposed to represent functional androgen-regulated enhancers (131). eRNAs have been shown to be functional in regulating AR target genes – for instance, an eRNA produced from the KLK3 enhancer (eKLK3) affects the expression of the important AR target genes KLK3, which encodes the PCa biomarker PSA (prostate-specific antigen), and the related KLK2 (26). Mechanistically, this is by promoting looping interactions between the KLK3 enhancer and the promoter for KLK2 (26). This eRNA may also cooperate with the MED1 protein to promote AR target gene expression (26).

**Figure 3:**
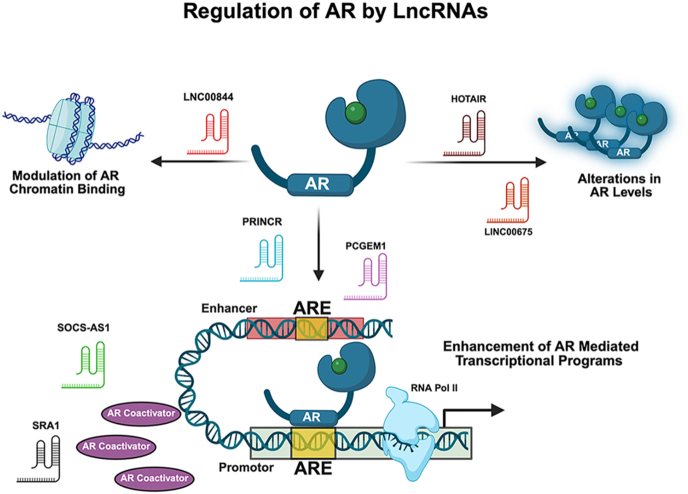
Examples of regulation of the AR by lncRNAs that affect the AR cistrome. lncRNAs may directly modulate the AR by altering AR binding to chromatin, modulating AR protein expression, impacting interactions with coactivators, and enhancing AR-mediated transcriptional activity.

Besides eRNAs, another class of noncoding RNAs, long noncoding RNAs (lncRNAs), can regulate AR-mediated transcriptional programs (Fig. 3). Although there is likely an overlap between the two classes, lncRNAs can, to some extent, be differentiated from eRNAs by their greater length and enhanced stability, transcription from independent promoters, and their frequent splicing as well as polyadenylation (133, 134). The lncRNAs can regulate AR transcriptional activity as part of the enhancer complex or indirectly by regulating AR levels. The lncRNAs that act by direct mechanisms include steroid receptor RNA activator 1 (SRA1), which was one of the first ncRNAs demonstrated to act as a transcriptional coactivator for nuclear receptors (135). SRA1 was found to enhance activity in a complex with the coactivator protein SRC, which promotes histone acetylation (135).

As other examples, the oncogenic lncRNAs PRINCR and PCGEM1 bind directly to the AR and enhance both ligand-dependent and independent AR activities (27). Their interaction with AR is sequential – PRINCR binds to acetylated AR on enhancers and recruits the methyltransferase DOT1L, which then methylates AR, resulting in binding of PCGEM1 (27). Besides the AR, both lncRNAs also interact with AR-V7, enhancer-associated histone modifications, and AR coactivators, such as CARM1 and GADD45α, and are involved in the formation of EPIs (27).

While this demonstrates a role for PRINCR and PCGEM1 lncRNAs in AR–enhancer complexes, it should be noted that a subsequent study was unable to replicate the AR- and histone-binding properties of these lncRNAs (136). However, SOCS-AS1 is another lncRNA that can modulate AR activity by binding to AR and recruiting coactivators (137). SOCS2-AS1 likely aids in the regulation of AR-induced and repressed genes by recruiting either histone acetylases, such as SRC1, or histone deacetylases (137).

LINC00844 also facilitates binding of AR to chromatin, and its depletion results in reprogramming of ARBS (49). This occurs without a direct physical interaction between LINC00844 and AR, suggesting that other intermediary factors may be involved (49). LINC00844 is a tumor suppressor that is largely lost in primary PCa and metastases. It mediates its tumor-suppressive effects by regulating AR-mediated expression of the tumor-suppressive N-Myc downstream regulated 1 (*NDRG1*) gene (49), thus inhibiting AR’s growth-suppressive transcriptional effects during tumor progression.

Finally, certain lncRNAs, such as HOTAIR and LINC00675, regulate module levels of the AR itself. HOTAIR has been shown to stabilize AR protein by binding to its N-terminal domain and preventing its interaction with MDM2, thus inhibiting subsequent ubiquitylation and degradation (138). Overexpression of this lncRNA leads to an increase in new ARBS and promotes AR activation in the absence of androgens (138). HOTAIR has also been shown to promote CRPC and enzalutamide resistance (138). LINC00675 similarly stabilizes AR by preventing its interaction with MDM2 and can also impact AR activity by stabilizing GATA2 transcripts (139). LINC00675 has been shown to be oncogenic in cell line models and is associated with higher expression in normal versus PCa tumor tissue as well as in CRPC versus primary PCa tissue (139).

### AR cistrome and the 3D genome

Long-range chromatin interactions are a key factor in AR-mediated gene expression, connecting distal regulatory elements to the promoters of target genes via chromatin loops (7, 30, 140). The formation of these three-dimensional loops is regulated by interactions between AR coregulatory factors on enhancers and target promoters (28). Enhancer–promoter interactions (EPIs) involving the AR were first described for the AR target gene KLK3 and shown to be mediated by an AR coactivation complex containing p160 proteins, CBP, p300, and pCAF (119, 141). ERG has been shown to colocalize with ARBS that form EPIs, with other coregulators such as FOXA1, EZH2, and the histone deacetylase HDAC3 also present at these sites (30). Cell cycle and apoptosis regulator 1 (CCAR1) has also been reported as an AR coactivator that is important for mediating AR-associated long-range interactions (142). CCAR1 is required for EPIs at the KLK3 gene, where it acts through recruiting GATA2 and stabilizing its interaction with AR (142).

Reprogramming of chromatin looping has been implicated in PCa progression – for example, looping between AR-bound enhancers and the promoter of the cell-cycle regulator gene *UBE2C* has been shown to drive expression of this gene in CRPC (31, 143). As another example, AR-dependent chromatin loop formation has been shown to repress expression of the *NR5A2* gene (144). Liver receptor homolog 1 (LRH-1), a nuclear receptor encoded by *NR5A2*, promotes intratumoral androgen synthesis and is upregulated during CRPC progression (144). This upregulation is proposed to be a result of the disruption of EPIs due to reduced AR signaling upon treatment with antiandrogens (144). AR coregulators FOXA1, GATA2, and MED1 colocalized on enhancers of the *UBE2C* gene, whereas EZH2, ERG, and HDACs were found to be colocalized on enhancers and promoters of the *NR5A2* gene (31, 144). More recently, AR stimulation was found to increase contact frequency between promoters of AR-upregulated genes and their looped AR-bound enhancers rather than cause a significant change in the number of loops (145). It has been suggested that enhancer-promoter loops may exist prior to AR stimulation, with AR binding resulting in recruitment of additional factors that increase contact frequency and, thus, increase gene expression (145).

## Clinical utility of the AR cistrome and targeting the AR–enhancer complex

**Figure 4:**
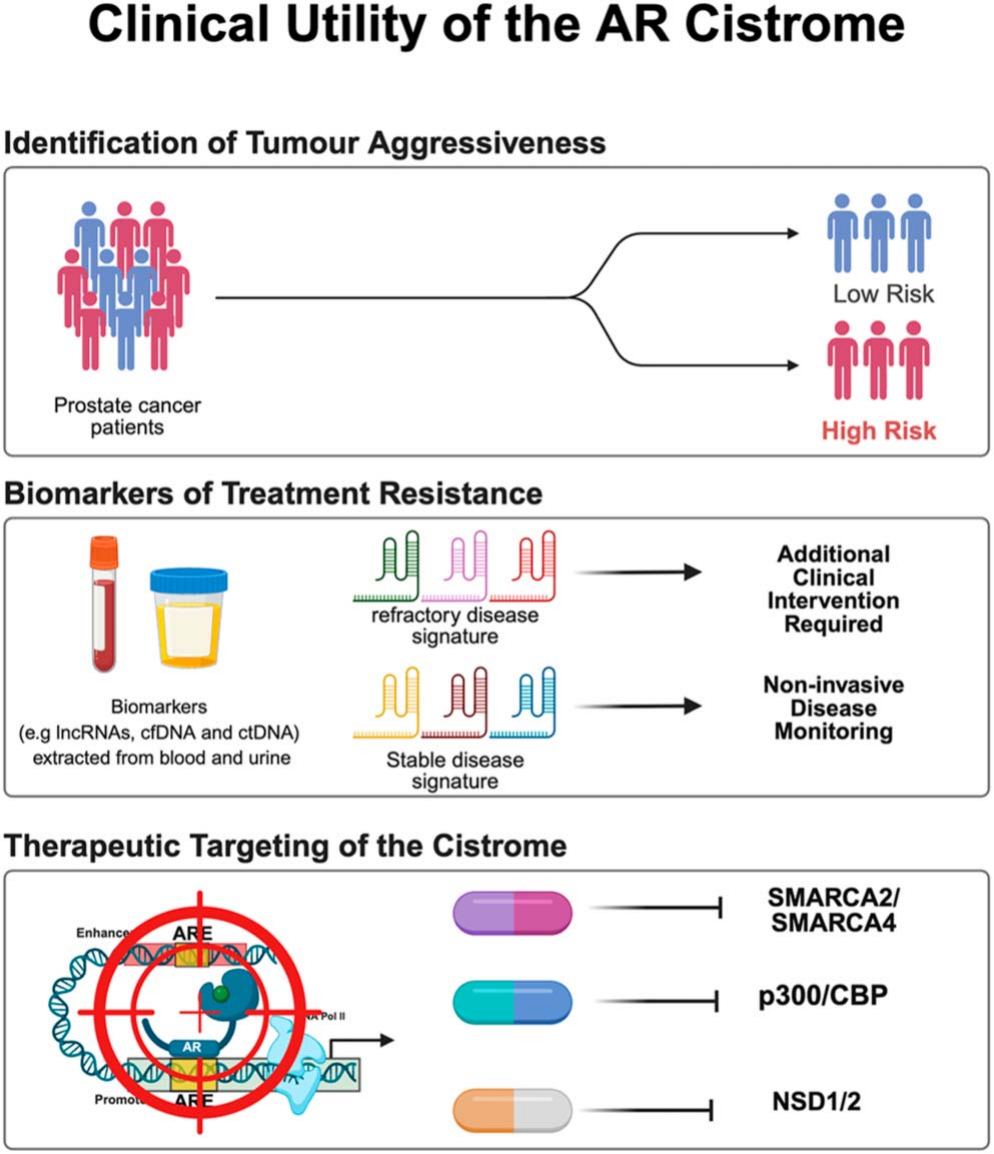
Illustration of the clinical utility of the AR cistrome in prostate cancer. Changes to the cistrome could potentially be used to identify tumor aggressiveness and stratify patients. Cistrome-associated ncRNA have the potential to be used as biomarkers, and components of the enhancer complex can be directly targeted therapeutically to disrupt oncogenic transcriptional programs. cfDNA, cell-free DNA; ctDNA, cell tumor DNA.

The changes in the AR cistrome observed during disease progression have the potential to be used for clinical purposes (Fig. 4) – for example, expression of gene sets linked with ARBS that are gained and lost in clinical states have been found to be prognostic for outcomes (146). Likewise, differences in AR–chromatin interactions between primary tumors and those with acquired therapy resistance have led to the generation of a gene signature for PCa outcome (57). AR cistromic data, when integrated with transcriptomic data and epigenetic profiles, can potentially also be used to distinguish PCa subtypes (147). Interestingly, AR cistromes show a significant amount of inter-individual heterogeneity in primary patient samples, which has implications for patient outcomes, with unique sites found to intersect with ARBS previously associated with metastases and poor outcomes (147).

AR-related alterations and ncRNA associated with the AR cistrome are of interest as clinical biomarkers, especially for use in liquid biopsies (148, 149, 150, 151). AR copy number gain, as detected in circulating tumor cells (CTCs) or cell-free DNA (cfDNA), has been associated with worse overall survival (84) and poor outcomes on treatment with ARPIs in patients with CRPC (148). Similarly, detection of AR-V7 in CTCs has been associated with poor outcomes for patients treated with ARPIs (152, 153). AR gene structural rearrangements detected in cfDNA, which likely arise from ecDNA, are associated with worse patient progression-free survival and overall survival (154). The potential utility of eRNAs as clinical biomarkers has also been evidenced in PCa, where a correlation was found between KLK3 mRNA and eRNAs in tumor samples (149). KLK3 mRNA itself is a potential prognostic biomarker for progression-free survival in CRPC (155). In addition to specific eRNAs, eRNA profiles are candidate biomarkers as they differ between states such as therapy resistance and sensitivity (156). lncRNAs are promising biomarkers in multiple disease conditions, including PCa. For example, prostate cancer antigen 3 (PCA3) is an lncRNA used as a biomarker to test for the presence of PCa, and MALAT1 has been explored as a diagnostic biomarker (157, 158), and those associated with the AR cistrome warrant further investigation in the context of PCa progression.

In terms of PCa treatment, disrupting enhancer complexes has the potential to be a useful strategy, and epigenetic coregulators are especially promising targets. Targeting these factors can interfere with oncogenic AR cistromes by compacting chromatin, inhibiting AR binding, and downregulating AR target genes (48, 109). Proteolysis targeting chimeras (PROTACs) have been developed in recent years that target the SWI/SNF subunits SMARCA2, SMARCA4, and PBRM1, as well as the histone modifiers NSD1/2 and p300/CBP (48, 109, 121). The SMARCA2/4 PROTAC AU-15330 has been shown to degrade SMARCCA2, SMARCA4, and PBRM1 (109). Although SWI/SNF complexes affect the expression of many genes, they are not universally required, and this drug appears to be selective toward prostate cancer cells in which growth is driven by AR and FOXA1, with less sensitivity observed in AR- and FOXA1-negative cells (109). AU-15330 also acts synergistically with enzalutamide to induce tumor regression in xenograft models (109). Anti-proliferative effects were not observed in normal and non-neoplastic prostate cells, and no systemic toxicity was reported in mice during xenograft experiments using this drug (109). The PROTAC against NSD1/2 (LLC0150) is also selective, with most cytotoxicity observed in hematological malignancies with activating NSD2 mutations and in AR-positive PCa cell lines (48). LLC0150 can also synergize with enzalutamide and is effective in enzalutamide-resistant PCa models (48). CDPD-409, which is a p300/CBP targeting PROTAC, is similarly more selective toward enhancer-driven, AR-positive PCa and has not been associated with systemic toxicity in xenograft models (121). Hence, there appear to be specific vulnerabilities associated with these complexes, despite their wide-ranging actions, that make targeting them a potentially feasible therapeutic strategy.

## Conclusion

While reactivated AR signaling has long been established as a causal factor in PCa progression, earlier work primarily focused on changes to the AR gene or protein. Reprogramming of the AR cistrome, with a shift toward usage of new oncogenic enhancers (i.e. neo-enhancers), is now known to play a key role in PCa progression. This phenomenon is a result of cooperation between AR, pioneer factors, and other components of the AR–enhancer complex. Pioneer factors and epigenetic coregulators appear to be important determinants of this, with several reports highlighting how changes in their levels can drive malignancy. More recently, ncRNAs have been shown to regulate AR cistromic changes, although this remains a relatively underexplored area of research. An increased understanding of these new contributors is beginning to allow identification of new clinical and therapeutic opportunities.

## Declaration of interest

The authors declare that there is no conflict of interest that could be perceived as prejudicing the impartiality of this review.

## Funding

This work was supported by funding from Prostate Cancer UK (TLD-CAF23-003 to RCF), the US Department of Defense (DOD) (W81XWH-22-1-0059 to RCF) and the Medical Research Council Doctoral Training Partnership (MR/N014103/1 to KKG).

## References

[1] Moore JE, Purcaro MJ, Pratt HE, et al. Expanded encyclopaedias of DNA elements in the human and mouse genomes. Nature 2020 583 699–710. (10.1038/s41586-020-2493-4) 32728249 PMC7410828

[2] Banerji J, Rusconi S & Schaffner W. Expression of a β-globin gene is enhanced by remote SV40 DNA sequences. Cell 1981 27 299–308. (10.1016/0092-8674(81)90413-x) 6277502

[3] Segert JA, Gisselbrecht SS & Bulyk ML. Transcriptional silencers: driving gene expression with the brakes on. Trends Genet 2021 37 514–527. (10.1016/j.tig.2021.02.002) 33712326 PMC8119328

[4] Banerji J, Olson L & Schaffner W. A lymphocyte-specific cellular enhancer is located downstream of the joining region in immunoglobulin heavy chain genes. Cell 1983 33 729–740. (10.1016/0092-8674(83)90015-6) 6409418

[5] Spitz F & Furlong EE. Transcription factors: from enhancer binding to developmental control. Nat Rev Genet 2012 13 613–626. (10.1038/nrg3207) 22868264

[6] Hnisz D, Abraham BJ, Lee TI, et al. Super-enhancers in the control of cell identity and disease. Cell 2013 155 934–947. (10.1016/j.cell.2013.09.053) 24119843 PMC3841062

[7] Massie CE, Lynch A, Ramos‐Montoya A, et al. The androgen receptor fuels prostate cancer by regulating central metabolism and biosynthesis. EMBO J 2011 30 2719–2733. (10.1038/emboj.2011.158) 21602788 PMC3155295

[8] Stelloo S, Bergman AM & Zwart W. Androgen receptor enhancer usage and the chromatin regulatory landscape in human prostate cancers. Endocr Relat Cancer 2019 26 R267–R285. (10.1530/erc-19-0032) 30865928

[9] Claessens F & Gewirth DT. DNA recognition by nuclear receptors. Essays Biochem 2004 40 59–72. (10.1042/bse0400059) 15242339

[10] Roche PJ, Hoare SA & Parker MG. A consensus DNA-binding site for the androgen receptor. Mol Endocrinol 1992 6 2229–2235. (10.1210/mend.6.12.1491700) 1491700

[11] Wilson S, Qi J & Filipp FV. Refinement of the androgen response element based on ChIP-Seq in androgen-insensitive and androgen-responsive prostate cancer cell lines. Sci Rep 2016 6 32611. (10.1038/srep32611) 27623747 PMC5021938

[12] Massie CE, Adryan B, Barbosa‐Morais NL, et al. New androgen receptor genomic targets show an interaction with the ETS1 transcription factor. EMBO Rep 2007 8 871–878. (10.1038/sj.embor.7401046) 17721441 PMC1950328

[13] Wasmuth EV, Hoover EA, Antar A, et al. Modulation of androgen receptor DNA binding activity through direct interaction with the ETS transcription factor ERG. Proc Natl Acad Sci U S A 2020 117 8584–8592. (10.1073/pnas.1922159117) 32220959 PMC7165421

[14] Kundaje A, Meuleman W, Ernst J, et al. Integrative analysis of 111 reference human epigenomes. Nature 2015 518 317–330. (10.1038/nature14248) 25693563 PMC4530010

[15] Barral A & Zaret KS. Pioneer factors: roles and their regulation in development. Trends Genet 2024 40 134–148. (10.1016/j.tig.2023.10.007) 37940484 PMC10873006

[16] Zaret KS & Carroll JS. Pioneer transcription factors: establishing competence for gene expression. Genes Dev 2011 25 2227–2241. (10.1101/gad.176826.111) 22056668 PMC3219227

[17] Hansen JL, Loell KJ & Cohen BA. A test of the pioneer factor hypothesis using ectopic liver gene activation. Elife 2022 11 e73358. (10.7554/elife.73358) 34984978 PMC8849321

[18] Pomerantz MM, Qiu X, Zhu Y, et al. Prostate cancer reactivates developmental epigenomic programs during metastatic progression. Nat Genet 2020 52 790–799. (10.1038/s41588-020-0664-8) 32690948 PMC10007911

[19] Heinlein CA & Chang C. Androgen receptor (AR) coregulators: an overview. Endocr Rev 2002 23 175–200. (10.1210/edrv.23.2.0460) 11943742

[20] Narlikar GJ, Sundaramoorthy R & Owen-Hughes T. Mechanisms and functions of ATP-dependent chromatin-remodeling enzymes. Cell 2013 154 490–503. (10.1016/j.cell.2013.07.011) 23911317 PMC3781322

[21] Bannister AJ & Kouzarides T. Regulation of chromatin by histone modifications. Cell Res 2011 21 381–395. (10.1038/cr.2011.22) 21321607 PMC3193420

[22] Greer EL & Shi Y. Histone methylation: a dynamic mark in health, disease and inheritance. Nat Rev Genet 2012 13 343–357. (10.1038/nrg3173) 22473383 PMC4073795

[23] Arrowsmith CH, Bountra C, Fish PV, et al. Epigenetic protein families: a new frontier for drug discovery. Nat Rev Drug Discov 2012 11 384–400. (10.1038/nrd3674) 22498752

[24] Franklin KA, Shields CE & Haynes KA. Beyond the marks: reader-effectors as drivers of epigenetics and chromatin engineering. Trends Biochem Sci 2022 47 417–432. (10.1016/j.tibs.2022.03.002) 35427480 PMC9074927

[25] Panigrahi A & O’Malley BW. Mechanisms of enhancer action: the known and the unknown. Genome Biol 2021 22 108. (10.1186/s13059-021-02322-1) 33858480 PMC8051032

[26] Hsieh C-L, Fei T, Chen Y, et al. Enhancer RNAs participate in androgen receptor-driven looping that selectively enhances gene activation. Proc Natl Acad Sci U S A 2014 111 7319–7324. (10.1073/pnas.1324151111) 24778216 PMC4034202

[27] Yang L, Lin C, Jin C, et al. lncRNA-dependent mechanisms of androgen-receptor-regulated gene activation programs. Nature 2013 500 598–602. (10.1038/nature12451) 23945587 PMC4034386

[28] Miele A & Dekker J. Long-range chromosomal interactions and gene regulation. Mol Biosyst 2008 4 1046–1057. (10.1039/b803580f) 18931780 PMC2653627

[29] Statello L, Guo C-J, Chen L-L, et al. Gene regulation by long non-coding RNAs and its biological functions. Nat Rev Mol Cell Biol 2021 22 96–118. (10.1038/s41580-020-00315-9) 33353982 PMC7754182

[30] Zhang Z, Chng KR, Lingadahalli S, et al. An AR-ERG transcriptional signature defined by long-range chromatin interactomes in prostate cancer cells. Genome Res 2019 29 223–235. (10.1101/gr.230243.117) 30606742 PMC6360806

[31] Wang Q, Li W, Zhang Y, et al. Androgen receptor regulates a distinct transcription program in androgen-independent prostate cancer. Cell 2009 138 245–256. (10.1016/j.cell.2009.04.056) 19632176 PMC2726827

[32] Panel PCPG. EAU PCa guidelines, 2025. (https://uroweb.org/guidelines/prostate-cancer/chapter/treatment)

[33] Harris WP, Mostaghel EA, Nelson PS, et al. Androgen deprivation therapy: progress in understanding mechanisms of resistance and optimizing androgen depletion. Nat Clin Pract Urol 2009 6 76–85. (10.1038/ncpuro1296) 19198621 PMC2981403

[34] Karantanos T, Corn PG & Thompson TC. Prostate cancer progression after androgen deprivation therapy: mechanisms of castrate resistance and novel therapeutic approaches. Oncogene 2013 32 5501–5511. (10.1038/onc.2013.206) 23752182 PMC3908870

[35] Scher HI, Beer TM, Higano CS, et al. Antitumour activity of MDV3100 in castration-resistant prostate cancer: a phase 1–2 study. Lancet 2010 375 1437–1446. (10.1016/s0140-6736(10)60172-9) 20398925 PMC2948179

[36] Ryan CJ, Smith MR, De Bono JS, et al. Abiraterone in metastatic prostate cancer without previous chemotherapy. N Engl J Med 2013 368 138–148. (10.1056/nejmoa1209096) 23228172 PMC3683570

[37] Estebanez-Perpina E, Bevan CL & McEwan IJ. Eighty years of targeting androgen receptor activity in prostate cancer: the fight goes on. Cancers 2021 13 509. (10.3390/cancers13030509) 33572755 PMC7865914

[38] Fizazi K, Foulon S, Carles J, et al. Abiraterone plus prednisone added to androgen deprivation therapy and docetaxel in de novo metastatic castration-sensitive prostate cancer (PEACE-1): a multicentre, open-label, randomised, phase 3 study with a 2 × 2 factorial design. Lancet 2022 399 1695–1707. (10.1016/s0140-6736(22)00367-1) 35405085

[39] Davis ID, Martin AJ, Stockler MR, et al. Enzalutamide with standard first-line therapy in metastatic prostate cancer. N Engl J Med 2019 381 121–131. (10.1056/nejmoa1903835) 31157964

[40] Smith MR, Hussain M, Saad F, et al. Darolutamide and survival in metastatic, hormone-sensitive prostate cancer. N Engl J Med 2022 386 1132–1142. (10.1056/nejmoa2119115) 35179323 PMC9844551

[41] Quigley DA, Dang HX, Zhao SG, et al. Genomic hallmarks and structural variation in metastatic prostate cancer. Cell 2018 174 758–769.e9. (10.1016/j.cell.2018.06.039) 30033370 PMC6425931

[42] Robinson D, Van Allen EM, Wu Y-M, et al. Integrative clinical genomics of advanced prostate cancer. Cell 2015 161 1215–1228. (10.1016/j.cell.2015.05.001) 26000489 PMC4484602

[43] Waltering KK, Urbanucci A & Visakorpi T. Androgen receptor (AR) aberrations in castration-resistant prostate cancer. Mol Cell Endocrinol 2012 360 38–43. (10.1016/j.mce.2011.12.019) 22245783

[44] Kallio HM, Hieta R, Latonen L, et al. Constitutively active androgen receptor splice variants AR-V3, AR-V7 and AR-V9 are co-expressed in castration-resistant prostate cancer metastases. Br J Cancer 2018 119 347–356. (10.1038/s41416-018-0172-0) 29988112 PMC6070921

[45] Sharp A, Coleman I, Yuan W, et al. Androgen receptor splice variant-7 expression emerges with castration resistance in prostate cancer. J Clin Invest 2019 129 192–208. (10.1172/jci122819) 30334814 PMC6307949

[46] Augello MA, Liu D, Deonarine LD, et al. CHD1 loss alters AR binding at lineage-specific enhancers and modulates distinct transcriptional programs to drive prostate tumorigenesis. Cancer Cell 2019 35 603–617.e8. (10.1016/j.ccell.2019.04.012) 30930119 PMC6467783

[47] Adams EJ, Karthaus WR, Hoover E, et al. FOXA1 mutations alter pioneering activity, differentiation and prostate cancer phenotypes. Nature 2019 571 408–412. (10.1038/s41586-019-1318-9) 31243370 PMC6661172

[48] Parolia A, Eyunni S, Verma BK, et al. NSD2 is a requisite subunit of the AR/FOXA1 neo-enhanceosome in promoting prostate tumorigenesis. Nat Genet 2024 56 1–12. (10.1038/s41588-024-01893-6) 39251788 PMC11525188

[49] Lingadahalli S, Jadhao S, Sung YY, et al. Novel lncRNA LINC00844 regulates prostate cancer cell migration and invasion through AR signaling. Mol Cancer Res 2018 16 1865–1878. (10.1158/1541-7786.mcr-18-0087) 30115758

[50] McNair C, Urbanucci A, Comstock CE, et al. Cell cycle-coupled expansion of AR activity promotes cancer progression. Oncogene 2017 36 1655–1668. (10.1038/onc.2016.334) 27669432 PMC5364060

[51] Pomerantz MM, Li F, Takeda DY, et al. The androgen receptor cistrome is extensively reprogrammed in human prostate tumorigenesis. Nat Genet 2015 47 1346–1351. (10.1038/ng.3419) 26457646 PMC4707683

[52] Nash C, Boufaied N, Badescu D, et al. Genome-wide analysis of androgen receptor binding and transcriptomic analysis in mesenchymal subsets during prostate development. Dis Model Mech 2019 12 dmm039297. (10.1242/dmm.039297) 31350272 PMC6679388

[53] Wang F & Koul HK. Androgen receptor (AR) cistrome in prostate differentiation and cancer progression. Am J Clin Exp Urol 2017 5 18. 29181434 PMC5698595

[54] Chen Z, Lan X, Thomas‐Ahner JM, et al. Agonist and antagonist switch DNA motifs recognized by human androgen receptor in prostate cancer. EMBO J 2015 34 502–516. (10.15252/embj.201490306) 25535248 PMC4331004

[55] Copeland BT, Du J, Pal SK, et al. Factors that influence the androgen receptor cistrome in benign and malignant prostate cells. Mol Oncol 2019 13 2616–2632. (10.1002/1878-0261.12572) 31520575 PMC6887583

[56] Sharma NL, Massie CE, Ramos-Montoya A, et al. The androgen receptor induces a distinct transcriptional program in castration-resistant prostate cancer in man. Cancer Cell 2013 23 35–47. (10.1016/j.ccr.2012.11.010) 23260764

[57] Stelloo S, Nevedomskaya E, van der Poel HG, et al. Androgen receptor profiling predicts prostate cancer outcome. EMBO Mol Med 2015 7 1450–1464. (10.15252/emmm.201505424) 26412853 PMC4644377

[58] Davies A, Nouruzi S, Ganguli D, et al. An androgen receptor switch underlies lineage infidelity in treatment-resistant prostate cancer. Nat Cell Biol 2021 23 1023–1034. (10.1038/s41556-021-00743-5) 34489572 PMC9012003

[59] Viswanathan SR, Ha G, Hoff AM, et al. Structural alterations driving castration-resistant prostate cancer revealed by linked-read genome sequencing. Cell 2018 174 433–447.e19. (10.1016/j.cell.2018.05.036) 29909985 PMC6046279

[60] Takeda DY, Spisák S, Seo J-H, et al. A somatically acquired enhancer of the androgen receptor is a noncoding driver in advanced prostate cancer. Cell 2018 174 422–432.e13. (10.1016/j.cell.2018.05.037) 29909987 PMC6046260

[61] Xiang RR, Lee S-A, Tyndall CF, et al. CRISPR screening identifies regulators of enhancer-mediated androgen receptor transcription in advanced prostate cancer. Cell Rep 2025 44 115312. (10.1016/j.celrep.2025.115312) 39954255 PMC11867844

[62] Morova T, McNeill DR, Lallous N, et al. Androgen receptor-binding sites are highly mutated in prostate cancer. Nat Commun 2020 11 832. (10.1038/s41467-020-14644-y) 32047165 PMC7012874

[63] Mazrooei P, Kron KJ, Zhu Y, et al. Cistrome partitioning reveals convergence of somatic mutations and risk variants on master transcription regulators in primary prostate tumors. Cancer Cell 2019 36 674–689.e6. (10.1016/j.ccell.2019.10.005) 31735626

[64] Huang C-CF, Lingadahalli S, Morova T, et al. Functional mapping of androgen receptor enhancer activity. Genome Biol 2021 22 149. (10.1186/s13059-021-02339-6) 33975627 PMC8112059

[65] Zhang X, Cowper-Sal lari R, Bailey SD, et al. Integrative functional genomics identifies an enhancer looping to the SOX9 gene disrupted by the 17q24.3 prostate cancer risk locus. Genome Res 2012 22 1437–1446. (10.1101/gr.135665.111) 22665440 PMC3409257

[66] Takayama K-I, Suzuki T, Fujimura T, et al. CtBP2 modulates the androgen receptor to promote prostate cancer progression. Cancer Res 2014 74 6542–6553. (10.1158/0008-5472.can-14-1030) 25228652

[67] Bu H, Narisu N, Schlick B, et al. Putative prostate cancer risk SNP in an androgen receptor‐binding site of the melanophilin gene illustrates enrichment of risk SNPs in androgen receptor target sites. Hum Mutat 2016 37 52–64. (10.1002/humu.22909) 26411452 PMC4715509

[68] Clinckemalie L, Spans L, Dubois V, et al. Androgen regulation of the TMPRSS2 gene and the effect of a SNP in an androgen response element. Mol Endocrinol 2013 27 2028–2040. (10.1210/me.2013-1098) 24109594 PMC5426606

[69] Ware KE, Garcia-Blanco MA, Armstrong AJ, et al. Biologic and clinical significance of androgen receptor variants in castration resistant prostate cancer. Endocr Relat Cancer 2014 21 T87–T103. (10.1530/erc-13-0470) 24859991 PMC4277180

[70] Chen CD, Welsbie DS, Tran C, et al. Molecular determinants of resistance to antiandrogen therapy. Nat Med 2004 10 33–39. (10.1038/nm972) 14702632

[71] Hu R, Dunn TA, Wei S, et al. Ligand-independent androgen receptor variants derived from splicing of cryptic exons signify hormone-refractory prostate cancer. Cancer Res 2009 69 16–22. (10.1158/0008-5472.can-08-2764) 19117982 PMC2614301

[72] Waltering KK, Helenius MA, Sahu B, et al. Increased expression of androgen receptor sensitizes prostate cancer cells to low levels of androgens. Cancer Res 2009 69 8141–8149. (10.1158/0008-5472.can-09-0919) 19808968

[73] Urbanucci A, Sahu B, Seppälä J, et al. Overexpression of androgen receptor enhances the binding of the receptor to the chromatin in prostate cancer. Oncogene 2012 31 2153–2163. (10.1038/onc.2011.401) 21909140

[74] Zhao SG, Bootsma M, Zhou S, et al. Integrated analyses highlight interactions between the three-dimensional genome and DNA, RNA and epigenomic alterations in metastatic prostate cancer. Nat Genet 2024 56 1689–1700. (10.1038/s41588-024-01826-3) 39020220 PMC11319208

[75] Zivanovic A, Miller JT, Munro SA, et al. Co-evolution of AR gene copy number and structural complexity in endocrine therapy resistant prostate cancer. NAR Cancer 2023 5 zcad045. (10.1093/narcan/zcad045) 37636316 PMC10448862

[76] Virtanen T, Kwan E, Parekh K, et al. Repertoire and clinical hierarchy of AR locus alterations in castration-resistant prostate cancer. Ann Oncol 2025 37 388–402. (10.1016/j.annonc.2025.10.1236) 41161480

[77] Zhu Y & Luo J. Regulation of androgen receptor variants in prostate cancer. Asian J Urol 2020 7 251–257. (10.1016/j.ajur.2020.01.001) 33024700 PMC7525062

[78] Antonarakis E, Armstrong A, Dehm S, et al. Androgen receptor variant-driven prostate cancer: clinical implications and therapeutic targeting. Prostate Cancer Prostatic Dis 2016 19 231–241. (10.1038/pcan.2016.17) 27184811 PMC5493501

[79] Lawrence MG, Keerthikumar S, Townley SL, et al. Reprogramming of androgen receptor activity in castration-resistant prostate cancer is shaped by truncated variants. Eur Urol Focus 2025 11 790–800. (10.1016/j.euf.2025.03.017) 40221372 PMC13180466

[80] Chen Z, Wu D, Thomas-Ahner JM, et al. Diverse AR-V7 cistromes in castration-resistant prostate cancer are governed by HoxB13. Proc Natl Acad Sci U S A 2018 115 6810–6815. (10.1073/pnas.1718811115) 29844167 PMC6042123

[81] Han D, Labaf M, Zhao Y, et al. Androgen receptor splice variants drive castration-resistant prostate cancer metastasis by activating distinct transcriptional programs. J Clin Investig 2024 134 e168649. (10.1172/JCI168649) 38687617 PMC11142739

[82] Cai L, Tsai Y-H, Wang P, et al. ZFX mediates non-canonical oncogenic functions of the androgen receptor splice variant 7 in castrate-resistant prostate cancer. Mol Cel 2018 72 341–354.e6. (10.1016/j.molcel.2018.08.029) PMC6214474 30270106

[83] Cato L, de Tribolet-Hardy J, Lee I, et al. ARv7 represses tumor-suppressor genes in castration-resistant prostate cancer. Cancer Cell 2019 35 401–413.e6. (10.1016/j.ccell.2019.01.008) 30773341 PMC7246081

[84] He Y, Lu J, Ye Z, et al. Androgen receptor splice variants bind to constitutively open chromatin and promote abiraterone-resistant growth of prostate cancer. Nucleic Acids Res 2018 46 1895–1911. (10.1093/nar/gkx1306) 29309643 PMC5829742

[85] Lu J, Lonergan PE, Nacusi LP, et al. The cistrome and gene signature of androgen receptor splice variants in castration resistant prostate cancer cells. J Urol 2015 193 690–698. (10.1016/j.juro.2014.08.043) 25132238 PMC4411637

[86] Basil P, Robertson MJ, Bingman WE III, et al. Cistrome and transcriptome analysis identifies unique androgen receptor (AR) and AR-V7 splice variant chromatin binding and transcriptional activities. Sci Rep 2022 12 5351. (10.1038/s41598-022-09371-x) 35354884 PMC8969163

[87] Jones D, Wade M, Nakjang S, et al. FOXA1 regulates androgen receptor variant activity in models of castrate-resistant prostate cancer. Oncotarget 2015 6 29782–29794. (10.18632/oncotarget.4927) 26336819 PMC4745762

[88] Xu K, Zhenhua JW, Groner AC, et al. EZH2 oncogenic activity in castration-resistant prostate cancer cells is polycomb independent. Science 2012 338 1465–1469. (10.1126/science.1227604) 23239736 PMC3625962

[89] Wang J, Park K-S, Yu X, et al. A cryptic transactivation domain of EZH2 binds AR and AR’s splice variant, promoting oncogene activation and tumorous transformation. Nucleic Acids Res 2022 50 10929–10946. (10.1093/nar/gkac861) 36300627 PMC9638897

[90] Lupien M, Eeckhoute J, Meyer CA, et al. FoxA1 translates epigenetic signatures into enhancer-driven lineage-specific transcription. Cell 2008 132 958–970. (10.1016/j.cell.2008.01.018) 18358809 PMC2323438

[91] Gao N, Ishii K, Mirosevich J, et al. Forkhead box A1 regulates prostate ductal morphogenesis and promotes epithelial cell maturation. Development 2005 132 3431–3443. (10.1242/dev.01917) 15987773

[92] Sahu B, Laakso M, Ovaska K, et al. Dual role of FoxA1 in androgen receptor binding to chromatin, androgen signalling and prostate cancer. EMBO J 2011 30 3962–3976. (10.1038/emboj.2011.328) 21915096 PMC3209787

[93] Wang D, Garcia-Bassets I, Benner C, et al. Reprogramming transcription by distinct classes of enhancers functionally defined by eRNA. Nature 2011 474 390–394. (10.1038/nature10006) 21572438 PMC3117022

[94] Jin H-J, Zhao JC, Wu L, et al. Cooperativity and equilibrium with FOXA1 define the androgen receptor transcriptional program. Nat Commun 2014 5 3972. (10.1038/ncomms4972) 24875621 PMC4088269

[95] Gao S, Chen S, Han D, et al. Forkhead domain mutations in FOXA1 drive prostate cancer progression. Cell Res 2019 29 770–772. (10.1038/s41422-019-0203-2) 31324883 PMC6796877

[96] Xu B, Song B, Lu X, et al. Altered chromatin recruitment by FOXA1 mutations promotes androgen independence and prostate cancer progression. Cell Res 2019 29 773–775. (10.1038/s41422-019-0204-1) 31324884 PMC6796844

[97] Parolia A, Cieslik M, Chu S-C, et al. Distinct structural classes of activating FOXA1 alterations in advanced prostate cancer. Nature 2019 571 413–418. (10.1038/s41586-019-1347-4) 31243372 PMC6661908

[98] Eyunni S, Mannan R, Zhang Y, et al. Divergent FOXA1 mutations drive prostate tumorigenesis and therapy-resistant cellular plasticity. Science 2025 389 eadv2367. (10.1126/science.adv2367) 40570057 PMC12326538

[99] Norris JD, Chang C-Y, Wittmann BM, et al. The homeodomain protein HOXB13 regulates the cellular response to androgens. Mol Cel 2009 36 405–416. (10.1016/j.molcel.2009.10.020) PMC2788777 19917249

[100] Ersoy-Fazlioglu B, Lingadahalli S, Altintas UB, et al. Distinct transcription factor interactions drive HOXB13 activity in different stages of prostate cancer. Proc Natl Acad Sci U S A 2025 122 e2500327122. (10.1073/pnas.2500327122) 41343677 PMC12704779

[101] Kim Y-R, Oh K-J, Park R-Y, et al. HOXB13 promotes androgen independent growth of LNCaP prostate cancer cells by the activation of E2F signaling. Mol Cancer 2010 9 124. (10.1186/1476-4598-9-124) 20504375 PMC2890607

[102] Wu D, Sunkel B, Chen Z, et al. Three-tiered role of the pioneer factor GATA2 in promoting androgen-dependent gene expression in prostate cancer. Nucleic Acids Res 2014 42 3607–3622. (10.1093/nar/gkt1382) 24423874 PMC3973339

[103] He B, Lanz RB, Fiskus W, et al. GATA2 facilitates steroid receptor coactivator recruitment to the androgen receptor complex. Proc Natl Acad Sci U S A 2014 111 18261–18266. (10.1073/pnas.1421415111) 25489091 PMC4280633

[104] Keo V, Lu X, Brea L, et al. A hierarchy of luminal transcription factors defines AR cistrome and is lost in neuroendocrine prostate cancer. Cancer Heterog Plast 2025 2 0008. (10.47248/chp2502020008) 41768226 PMC12945349

[105] Kanayama M, Chen Y, Rabizadeh D, et al. Clinical and functional analyses of an African-ancestry gain-of-function HOXB13 variant implicated in aggressive prostate cancer. Eur Urol Oncol 2024 7 751–759. (10.1016/j.euo.2023.09.012) 37806842 PMC12848658

[106] Ewing CM, Ray AM, Lange EM, et al. Germline mutations in HOXB13 and prostate-cancer risk. N Engl J Med 2012 366 141–149. (10.1056/nejmoa1110000) 22236224 PMC3779870

[107] Chaytor L, Simcock M, Nakjang S, et al. The pioneering role of GATA2 in androgen receptor variant regulation is controlled by bromodomain and extraterminal proteins in castrate-resistant prostate cancer. Mol Cancer Res 2019 17 1264–1278. (10.1158/1541-7786.mcr-18-1231) 30833300

[108] Launonen K-M, Paakinaho V, Sigismondo G, et al. Chromatin-directed proteomics-identified network of endogenous androgen receptor in prostate cancer cells. Oncogene 2021 40 4567–4579. (10.1038/s41388-021-01887-2) 34127815 PMC8266679

[109] Xiao L, Parolia A, Qiao Y, et al. Targeting SWI/SNF ATPases in enhancer-addicted prostate cancer. Nature 2022 601 434–439. (10.1038/s41586-021-04246-z) 34937944 PMC8770127

[110] Varambally S, Dhanasekaran SM, Zhou M, et al. The polycomb group protein EZH2 is involved in progression of prostate cancer. Nature 2002 419 624–629. (10.1038/nature01075) 12374981

[111] Park SH, Fong K-W, Mong E, et al. Going beyond polycomb: EZH2 functions in prostate cancer. Oncogene 2021 40 5788–5798. (10.1038/s41388-021-01982-4) 34349243 PMC8487936

[112] Labaf M, Li M, Ting L, et al. Increased AR expression in castration-resistant prostate cancer rapidly induces AR signaling reprogramming with the collaboration of EZH2. Front Oncol 2022 12 1021845. (10.3389/fonc.2022.1021845) 36408179 PMC9669968

[113] Aytes A, Giacobbe A, Mitrofanova A, et al. NSD2 is a conserved driver of metastatic prostate cancer progression. Nat Commun 2018 9 5201. (10.1038/s41467-018-07511-4) 30518758 PMC6281610

[114] Bianco-Miotto T, Chiam K, Buchanan G, et al. Global levels of specific histone modifications and an epigenetic gene signature predict prostate cancer progression and development. Cancer Epidemiol Biomarkers Prev 2010 19 2611–2622. (10.1158/1055-9965.epi-10-0555) 20841388

[115] Tang S, Sethunath V, Metaferia NY, et al. A genome-scale CRISPR screen reveals PRMT1 as a critical regulator of androgen receptor signaling in prostate cancer. Cell Rep 2022 38 110417. (10.1016/j.celrep.2022.110417) 35196489 PMC9036938

[116] Welti J, Sharp A, Brooks N, et al. Targeting the p300/CBP axis in lethal prostate cancer. Cancer Discov 2021 11 1118–1137. (10.1158/2159-8290.cd-20-0751) 33431496 PMC8102310

[117] Agoulnik IU, Vaid A, Nakka M, et al. Androgens modulate expression of transcription intermediary factor 2, an androgen receptor coactivator whose expression level correlates with early biochemical recurrence in prostate cancer. Cancer Res 2006 66 10594–10602. (10.1158/0008-5472.can-06-1023) 17079484

[118] Halkidou K, Gnanapragasam VJ, Mehta PB, et al. Expression of Tip60, an androgen receptor coactivator, and its role in prostate cancer development. Oncogene 2003 22 2466–2477. (10.1038/sj.onc.1206342) 12717424

[119] Shang Y, Myers M & Brown M. Formation of the androgen receptor transcription complex. Mol Cel 2002 9 601–610. (10.1016/s1097-2765(02)00471-9) 11931767

[120] Jin L, Garcia J, Chan E, et al. Therapeutic targeting of the CBP/p300 bromodomain blocks the growth of castration-resistant prostate cancer. Cancer Res 2017 77 5564–5575. (10.1158/0008-5472.can-17-0314) 28819026

[121] Luo J, Chen Z, Qiao Y, et al. Targeting histone H2B acetylated enhanceosomes via p300/CBP degradation in prostate cancer. Nat Genet 2025 57 2468–2481. (10.1038/s41588-025-02336-6) 41044247 PMC12513837

[122] Groner AC, Cato L, de Tribolet-Hardy J, et al. TRIM24 is an oncogenic transcriptional activator in prostate cancer. Cancer Cell 2016 29 846–858. (10.1016/j.ccell.2016.04.012) 27238081 PMC5124371

[123] Qian C, Li D & Chen Y. ETS factors in prostate cancer. Cancer Lett 2022 530 181–189. (10.1016/j.canlet.2022.01.009) 35033589 PMC8832285

[124] Nicholas TR, Strittmatter BG & Hollenhorst PC. Oncogenic ETS factors in prostate cancer. In Prostate Cancer: Cellular and Genetic Mechanisms of Disease Development and Progression, pp 409–436. Eds SM Dehm & JT Donald. Cham: Springer, 2019. (10.1007/978-3-030-32656-2_18) 31900919

[125] Tomlins SA, Rhodes DR, Perner S, et al. Recurrent fusion of TMPRSS2 and ETS transcription factor genes in prostate cancer. Science 2005 310 644–648. (10.1126/science.1117679) 16254181

[126] Chen Y, Chi P, Rockowitz S, et al. ETS factors reprogram the androgen receptor cistrome and prime prostate tumorigenesis in response to PTEN loss. Nat Med 2013 19 1023–1029. (10.1038/nm.3216) 23817021 PMC3737318

[127] Rasool R, Natesan R, Deng Q, et al. CDK7 inhibition suppresses castration-resistant prostate cancer through MED1 inactivation. Cancer Discov 2019 9 1538–1555. (10.1158/2159-8290.cd-19-0189) 31466944 PMC7202356

[128] De Santa F, Barozzi I, Meitton F, et al. A large fraction of extragenic RNA Pol II transcription sites overlap enhancers. PLoS Biol 2010 8 e1000384. (10.1371/journal.pbio.1000384) 20485488 PMC2867938

[129] Kim TK, Hemberg M, Gray JM, et al. Widespread transcription at neuronal activity-regulated enhancers. Nature 2010 465 182–187. (10.1038/nature09033) 20393465 PMC3020079

[130] Koch F, Fenouil R, Gut M, et al. Transcription initiation platforms and GTF recruitment at tissue-specific enhancers and promoters. Nat Struct Mol Biol 2011 18 956–963. (10.1038/nsmb.2085) 21765417

[131] Toropainen S, Niskanen EA, Malinen M, et al. Global analysis of transcription in castration-resistant prostate cancer cells uncovers active enhancers and direct androgen receptor targets. Sci Rep 2016 6 33510. (10.1038/srep33510) 27641228 PMC5027586

[132] Zhao Y, Wang L, Ren S, et al. Activation of P-TEFb by androgen receptor-regulated enhancer RNAs in castration-resistant prostate cancer. Cell Rep 2016 15 599–610. (10.1016/j.celrep.2016.03.038) 27068475 PMC5395199

[133] Espinosa JM. Revisiting lncRNAs: how do you know yours is not an eRNA? Mol Cel 2016 62 1–2. (10.1016/j.molcel.2016.03.022) 27058782

[134] Han Z & Li W. Enhancer RNA: what we know and what we can achieve. Cell Prolif 2022 55 e13202. (10.1111/cpr.13202) 35170113 PMC9055912

[135] Lanz RB, McKenna NJ, Onate SA, et al. A steroid receptor coactivator, SRA, functions as an RNA and is present in an SRC-1 complex. Cell 1999 97 17–27. (10.1016/s0092-8674(00)80711-4) 10199399

[136] Prensner JR, Sahu A, Iyer MK, et al. The lncRNAs PCGEM1 and PRNCR1 are not implicated in castration resistant prostate cancer. Oncotarget 2014 5 1434–1438. (10.18632/oncotarget.1846) 24727738 PMC4039221

[137] Misawa A, Takayama K-I, Urano T, et al. Androgen-induced long noncoding RNA (lncRNA) SOCS2-AS1 promotes cell growth and inhibits apoptosis in prostate cancer cells. J Biol Chem 2016 291 17861–17880. (10.1074/jbc.m116.718536) 27342777 PMC5016176

[138] Zhang A, Zhao JC, Kim J, et al. LncRNA HOTAIR enhances the androgen-receptor-mediated transcriptional program and drives castration-resistant prostate cancer. Cell Rep 2015 13 209–221. (10.1016/j.celrep.2015.08.069) 26411689 PMC4757469

[139] Yao M, Shi X, Li Y, et al. LINC00675 activates androgen receptor axis signaling pathway to promote castration-resistant prostate cancer progression. Cell Death Dis 2020 11 638. (10.1038/s41419-020-02856-5) 32801300 PMC7429955

[140] Wu D, Zhang C, Shen Y, et al. Androgen receptor-driven chromatin looping in prostate cancer. Trends Endocrinol Metab 2011 22 474–480. (10.1016/j.tem.2011.07.006) 21889355 PMC3229688

[141] Wang Q, Carroll JS & Brown M. Spatial and temporal recruitment of androgen receptor and its coactivators involves chromosomal looping and polymerase tracking. Mol Cel 2005 19 631–642. (10.1016/j.molcel.2005.07.018) 16137620

[142] Seo W-Y, Jeong BC, Yu EJ, et al. CCAR1 promotes chromatin loading of androgen receptor (AR) transcription complex by stabilizing the association between AR and GATA2. Nucleic Acids Res 2013 41 8526–8536. (10.1093/nar/gkt644) 23887938 PMC3794601

[143] Chen Z, Zhang C, Wu D, et al. Phospho‐MED1‐enhanced UBE2C locus looping drives castration‐resistant prostate cancer growth. EMBO J 2011 30 2405–2419. (10.1038/emboj.2011.154) 21556051 PMC3116285

[144] You W, Gao T, Li H, et al. Androgen receptor acts as the transcriptional repressor of the nuclear receptor LRH-1 via the androgen-driven chromatin looping conformation in prostate cancer. Genes Dis 2025 13 101903. (10.1016/j.gendis.2025.101903) 42004222 PMC13090603

[145] Altıntaş UB, Seo J-H, Giambartolomei C, et al. Decoding the epigenetics and chromatin loop dynamics of androgen receptor-mediated transcription. Nat Commun 2024 15 9494. (10.1038/s41467-024-53758-5) 39489778 PMC11532539

[146] Severson T, Qiu X, Alshalalfa M, et al. Androgen receptor reprogramming demarcates prognostic, context-dependent gene sets in primary and metastatic prostate cancer. Clin Epigenet 2022 14 60. (10.1186/s13148-022-01278-8) PMC9069737 35509021

[147] Kneppers J, Severson TM, Siefert JC, et al. Extensive androgen receptor enhancer heterogeneity in primary prostate cancers underlies transcriptional diversity and metastatic potential. Nat Commun 2022 13 7367. (10.1038/s41467-022-35135-2) 36450752 PMC9712620

[148] Conteduca V, Wetterskog D, Sharabiani MT, et al. Androgen receptor gene status in plasma DNA associates with worse outcome on enzalutamide or abiraterone for castration-resistant prostate cancer: a multi-institution correlative biomarker study. Ann Oncol 2017 28 1508–1516. (10.1093/annonc/mdx155) 28472366 PMC5834043

[149] Nishimura K, Mori J, Sawada T, et al. Profiling of androgen-dependent enhancer RNAs expression in human prostate tumors: search for malignancy transition markers. Res Rep Urol 2021 13 705–713. (10.2147/rru.s328661) 34549035 PMC8449685

[150] Kwan EM & Wyatt AW. Androgen receptor genomic alterations and treatment resistance in metastatic prostate cancer. Prostate 2022 82 S25–S36. (10.1002/pros.24356) 35657159

[151] Luo J. Non-invasive actionable biomarkers for metastatic prostate cancer. Asian J Urol 2016 3 170–176. (10.1016/j.ajur.2016.09.003) 29264186 PMC5730867

[152] Antonarakis ES, Lu C, Luber B, et al. Clinical significance of androgen receptor splice variant-7 mRNA detection in circulating tumor cells of men with metastatic castration-resistant prostate cancer treated with first-and second-line abiraterone and enzalutamide. J Clin Oncol 2017 35 2149–2156. (10.1200/jco.2016.70.1961) 28384066 PMC5493048

[153] Armstrong AJ, Halabi S, Luo J, et al. Prospective multicenter validation of androgen receptor splice variant 7 and hormone therapy resistance in high-risk castration-resistant prostate cancer: the PROPHECY study. J Clin Oncol 2019 37 1120–1129. (10.1200/jco.18.01731) 30865549 PMC6494355

[154] Knutson TP, Luo B, Kobilka A, et al. AR alterations inform circulating tumor DNA detection in metastatic castration resistant prostate cancer patients. Nat Commun 2024 15 10648. (10.1038/s41467-024-54847-1) 39663356 PMC11634963

[155] Boerrigter E, Benoist GE, van Oort IM, et al. Liquid biopsy reveals KLK3 mRNA as a prognostic marker for progression free survival in patients with metastatic castration‐resistant prostate cancer undergoing first‐line abiraterone acetate and prednisone treatment. Mol Oncol 2021 15 2453–2465. (10.1002/1878-0261.12933) 33650292 PMC8410566

[156] Zhao J, Zhao Y, Wang L, et al. Alterations of androgen receptor-regulated enhancer RNAs (eRNAs) contribute to enzalutamide resistance in castration-resistant prostate cancer. Oncotarget 2016 7 38551. (10.18632/oncotarget.9535) 27221037 PMC5122410

[157] Wang F, Ren S, Chen R, et al. Development and prospective multicenter evaluation of the long noncoding RNA MALAT-1 as a diagnostic urinary biomarker for prostate cancer. Oncotarget 2014 5 11091. (10.18632/oncotarget.2691) 25526029 PMC4294360

[158] Cui Y, Cao W, Li Q, et al. Evaluation of prostate cancer antigen 3 for detecting prostate cancer: a systematic review and meta-analysis. Sci Rep 2016 6 25776. (10.1038/srep25776) 27161545 PMC4861967

